# Motion Analytics of Trapezius Muscle Activity in an 18-Year-Old Female with Extended Upper Brachial Plexus Birth Palsy

**DOI:** 10.1055/s-0041-1731748

**Published:** 2021-10-26

**Authors:** Jasmine J. Lin, Gromit Y.Y. Chan, Cláudio T. Silva, Luis G. Nonato, Preeti Raghavan, Aleksandra McGrath, Alice Chu

**Affiliations:** 1 Department of Orthopaedics, Rutgers New Jersey Medical School, Newark, New Jersey, United States; 2 Tandon School of Engineering, New York University, New York, United States; 3 Instituto de Ciências Matemáticas e de Computação (ICMC), University of São Paulo, São Paulo, Brazil; 4 Department of Physical Medicine and Rehabilitation and Neurology, Johns Hopkins University School of Medicine, Baltimore, Maryland, United States; 5 Department of Clinical Sciences, Umeå University, Umeå, Sweden

**Keywords:** brachial plexus, motion analysis, birth palsy, nerve transfer, trapezius, motion browser

## Abstract

**Background** The trapezius muscle is often utilized as a muscle or nerve donor for repairing shoulder function in those with brachial plexus birth palsy (BPBP). To evaluate the native role of the trapezius in the affected limb, we demonstrate use of the Motion Browser, a novel visual analytics system to assess an adolescent with BPBP.

**Method** An 18-year-old female with extended upper trunk (C5–6–7) BPBP underwent bilateral upper extremity three-dimensional motion analysis with Motion Browser. Surface electromyography (EMG) from eight muscles in each limb which was recorded during six upper extremity movements, distinguishing between upper trapezius (UT) and lower trapezius (LT). The Motion Browser calculated active range of motion (AROM), compiled the EMG data into measures of muscle activity, and displayed the results in charts.

**Results** All movements, excluding shoulder abduction, had similar AROM in affected and unaffected limbs. In the unaffected limb, LT was more active in proximal movements of shoulder abduction, and shoulder external and internal rotations. In the affected limb, LT was more active in distal movements of forearm pronation and supination; UT was more active in shoulder abduction.

**Conclusion** In this female with BPBP, Motion Browser demonstrated that the native LT in the affected limb contributed to distal movements. Her results suggest that sacrificing her trapezius as a muscle or nerve donor may affect her distal functionality. Clinicians should exercise caution when considering nerve transfers in children with BPBP and consider individualized assessment of functionality before pursuing surgery.

## Introduction

For brachial plexus surgeons, there are few areas more intriguing than the dynamics of how children with brachial plexus birth palsy (BPBP) use their affected arm. 1 2 3 Functionality of the affected arm is not well understood because the injury occurs in the perinatal period and the disease transforms as the child grows. Nerve and muscle weakness develop over time, as well as changes to muscle, joint, bone, and compensatory muscle recruitment. 4

One interesting question in children with BPBP is whether the upper or lower trapezius (UT and LT, respectively) muscles are functionally active during upper extremity use. It is intriguing because there are three ways in which an innervated trapezius can be utilized in children with BPBP, leaving it in situ, denervation and use of the trapezius' spinal accessory nerve (SAN) for distal nerve transfers, or using it directly as a muscle transfer. 5 6 7 The prevailing surgical technique of treating BPBP addresses a compromised suprascapular nerve through a posterior approach of transferring the lower portion of the SAN which innervates the LT and leaving innervation of the UT intact. 8 9

Because there are no studies looking at the natural contribution of the trapezius in children with BPBP, the morbidity of harvesting the SAN is largely unknown. It is known that normal children who sustain iatrogenic SAN injury have limited shoulder abduction of 70 to 90 degrees, and that divisions of trapezius muscles each have diverse roles in scapulothoracic movements that contribute to glenohumeral stability. 10 11 12

To better understand the functional role of the trapezius in children with BPBP, this report presents pilot data on UT and LT activity in an 18-year-old female with extended upper brachial plexus (C5–6–7) birth palsy. It is the first of its kind to demonstrate use of the Motion Browser, a novel visual analytics system developed at New York University. Motion Browser is a tool that synchronizes video recordings with kinematic and EMG data for assessment of muscle groups. 13

## Methods

An 18-year-old female with right-sided extended upper trunk (C5–6–7) BPBP, Mallet score of 22, and a prior anterior shoulder release underwent bilateral upper extremity analysis with the Motion Browser. She had no prior nerve transfer surgery and was free of neurological or metabolic impairment beyond BPBP. Examination at time of presentation confirmed intact trapezius function; the only physical limitation was right shoulder motion. She had mild internal shoulder rotation, elbow flexion contracture deformity, and weaker wrist flexion compared with the contralateral side, but was greater than the British Medical Research Council (BMRC), grade 3 in all tested motions. In this institutional review board (IRB) approved study at New York University, the patient was evaluated with simultaneous three-dimensional motion analysis, 16-channel electromyography (EMG), and video monitoring.

Data were recorded for eight muscles in each upper limb as follows: (1) biceps, (2) triceps, (3) pronator teres, (4) pronator quadratus, (5) UT, (6) LT, (7) flexor digitorum superficialis, and (8) extensor digitorum communis. The following six upper extremity movements were performed: (1) shoulder abduction, (2) shoulder external rotation, (3) shoulder internal rotation, (4) elbow flexion, (5) forearm pronation, and (6) forearm supination. Movements were tested individually in each limb. Only one recording for each limb was assessed per movement. Shoulder external/internal rotation was performed with the arm in the adducted position. Nonshoulder movements of elbow flexion and forearm pronation/supination were called distal movements. EMG, kinematic data, and video recordings were processed through the Motion Browser. 13

**Fig. 1:**
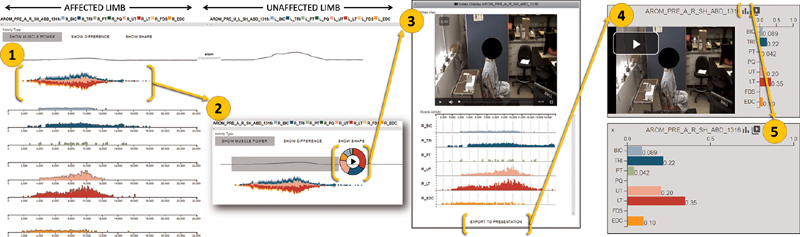
The Motion Browser data extraction method. 1. The Motion Browser displays the synchronized video, kinematics, and electromyography (EMG) data for the patient's affected and unaffected limbs for each movement performed. 2. The user selected segments of interest based on examining the video clips to fit selection criteria. Selected segments included all EMG data corresponding to the movement recorded in that video segment. 3. The Motion Browser isolated the motion data for that segment of interest. 4. The Motion Brower displayed a bar chart depicting the fraction of each muscle's activation relative to the total measured activity in the movement in the segment of interest. 5. Individual bar values for muscle activation were recorded for results analysis.

Data were selected in the Motion Browser, starting from the auditory tone that prompted the patient to initiate motion and ending when the arm returned to the resting position. Twelve-movement segments were collected, six movements per limb. Within each movement, maximal active motion and muscle activity patterns were recorded. Fig. 1 displays the data extraction steps.

The Motion Browser calculated active range of motion (AROM) as the angular displacement (in degrees) between the highest point of upper limb placement and the lowest point of the patient's resting position. Pronation and supination were measured with supination scored as negative. The similarity ratio between the maximum AROM in the affected versus the unaffected limb was calculated as a percentage. If the patient produced more voluntary motion in the affected than in the unaffected limb, a ratio of greater than 100% was calculated.

The Motion Browser compiled the EMG data throughout the segment into measures of muscle activity by the root-mean-square (RMS) envelope and displayed the results of the eight muscle groups in bar charts. 11 Each bar value demonstrated the fraction of muscle activity relative to the total measured activity throughout the movement. The higher the bar value, the higher the individual muscle activity. Muscle activity for UT and LT was isolated for assessment.

## Results

### Voluntary Active Range of Motion Was Similar in Both Affected and Unaffected Limbs

Similarity of the voluntary affected/unaffected maximal AROM is shown in Table 1 . All movements excluding shoulder abduction had a similarity ratio (affected/unaffected) greater than or equal to 90%. Only one movement, shoulder abduction, had an affected/unaffected ratio <90%, at 60.81%.

**Table 1:** Results for comparing the ratio of angular displacement of active motion between affected and unaffected limbs in upper extremity movements (cm)

Movement	Affected	Unaffected	Similarity ratio (affected/unaffected) %
Shoulder abduction	74.8	123	60.81
Shoulder external rotation	57.6	60.6	95.05
Shoulder internal rotation	67.4	64	105.31
Elbow flexion	162	152	106.58
Forearm pronation	128	142	90.14
Forearm supination	113	123	91.87

### Lower Trapezius Was More Active in the Affected Limb during Distal Movements

Trapezius muscle activity shown in Table 2 displays the percentage of EMG activity contributed by UT and LT in all movements in each limb. UT had no activity in affected or unaffected limbs in shoulder external rotation, shoulder internal rotation, elbow flexion, forearm pronation, and forearm supination. UT accounted for 20.0% of muscle activity in the affected limb during shoulder abduction and 0.5% in the unaffected during shoulder abduction.

**Table 2:** Percentage of EMG activity contributed by the UT and LT in the six tested movements in both affected and unaffected limbs

UT activity (%)		
Movement	Affected	Unaffected
Shoulder abduction	20.0	0.5
Shoulder external rotation	0.0	0.0
Shoulder internal rotation	0.0	0.0
Elbow flexion	0.0	0.0
Forearm pronation	0.0	0.0
Forearm supination	0.0	0.0
**LT activity (%)**		
**Movement**	**Affected**	**Unaffected**
Shoulder abduction	35.0	85.0
Shoulder external rotation	6.0	30.0
Shoulder internal rotation	7.9	61.0
Elbow flexion	35.0	17.0
Forearm pronation	59.0	7.0
Forearm supination	87.0	23.0

Abbreviations: EMG, electromyography; LT, lower trapezius; UT, upper trapezius.

LT was most active (>50%) in the unaffected limb during shoulder abduction (85.0%) and shoulder internal rotation (61.0%). It was most active (>50%) in the affected limb during the distal movements of forearm pronation (59.0%) and forearm supination (87.0%). Activity in the unaffected limb was more than that in the affected limb during shoulder abduction (85.0% unaffected and 35.0% affected), shoulder external rotation (30.0% unaffected and 6.0% affected), and shoulder internal rotation (61.0% unaffected and 8.0% affected). Activity in the affected limb was more than that in the unaffected during elbow flexion (35.0% affected and 17.0% unaffected), forearm pronation (59.0% affected and 7.0% unaffected), and forearm supination (87.0% affected and 23.0% unaffected).

## Discussion

The effect of the trapezius muscle, while known to be vital for upper limb function in healthy children, 11 has never been studied in children/adolescents with BPBP. Over the past decade, studies have discussed the transfer of the trapezius' SAN for a compromised suprascapular nerve in treating BPBP. 14 15 16 However, a necessary prerequisite to perform a nerve transfer is the consensus that the donor nerve is expendable and will not downgrade the patient's upper extremity function. 17 18 Morbidity of trapezius innervation or transfer should first be determined based on knowledge about its function in the natural state of injury.

Most authors who study upper extremity function in neurological diseases utilize kinematics or combined EMG analysis. 18 19 20 21 The Motion Browser is unique because it expands upon that methodology by synchronizing video recordings with kinematic and EMG data, thus corresponding information at each time point can be isolated. EMG data are normalized by comparing muscle activity within the limb, and the browser displays percentages of each muscle activity. 13

This patient's Motion Browser results suggest that her LT is more active in the affected limb during the movements of forearm pronation (59.0%) and supination (87.0%) than in the unaffected (17.0 and 23.0%, respectively). The AROM of the affected limb when compared with the unaffected during these movements had a similarity ratio of >90.0%. In these movements that are not typically expected to provoke trapezius activity, the muscle likely plays a part in isokinetic stabilization of the scapulothoracic region. 10 11 12 Conversely, for UT, activity was nearly zero for all movements in the affected limb except for shoulder abduction (20.0%) and minimally active in the unaffected for all movements. In the video footage, the patient appeared to lift the affected shoulder to complete shoulder abduction. UT activation can thus be attributed to compensating for the abduction deficit, a sequela of the brachial plexus injury.

This report demonstrates the use of the Motion Browser for analysis of the trapezius muscle in an adolescent with BPBP. This patient's results in the affected limb showed LT active a decreased amount during functional shoulder and elbow motion, but relatively active during the most distal movements of forearm supination and pronation. Results suggested that in this specific patient, transferring the SAN could potentially compromise distal function at the expense of shoulder restoration.

## Limitations

Limitations of the Motion Browser include the constraints of data collection. This setup is expensive because analyses need to be completed in a laboratory with adequate equipment. Furthermore, EMG signals are collected through surface electrodes, which are prone to background noise and difficulty with normalization.

## Conclusion

In conclusion, clinicians may wish to exercise caution when considering the current surgical treatment of the transfer of the SAN for the suprascapular nerve. Studies in patients who did and did not receive nerve transfer have found no significant differences in external rotation of the arm 22 or in recovery of ROM or strength 23 ; postsurgical follow-ups have found no trapezius atrophy/weakness. 24 Motion systems, like the Motion Browser, can help clinicians better evaluate the native use of the trapezius muscle in BPBP patients before considering surgery.

## References

[1] Abzug J M Kozin S H Evaluation and management of brachial plexus birth palsy Orthop Clin North Am 2014 45 02 225 232 24684916 10.1016/j.ocl.2013.12.004

[2] Duff S V Dayanidhi S Kozin S H Asymmetrical shoulder kinematics in children with brachial plexus birth palsy Clin Biomech (Bristol, Avon) 2007 22 06 630 638 10.1016/j.clinbiomech.2007.02.002 17412464

[3] Waters P M Comparison of the natural history, the outcome of microsurgical repair, and the outcome of operative reconstruction in brachial plexus birth palsy J Bone Joint Surg Am 1999 81 05 649 659 10360693 10.2106/00004623-199905000-00006

[4] Min W Price A E Alfonso I Ramos L Grossman J A Hypoplasia of the trapezius and history of ipsilateral transient neonatal brachial plexus palsy Pediatr Neurol 2011 44 03 225 228 21310341 10.1016/j.pediatrneurol.2010.10.008

[5] Colbert S H Mackinnon S Posterior approach for double nerve transfer for restoration of shoulder function in upper brachial plexus palsy Hand (N Y) 2006 1 02 71 77 18780028 10.1007/s11552-006-9004-4 PMC2526027

[6] Kawabata H Kawai H Masatomi T Yasui N Accessory nerve neurotization in infants with brachial plexus birth palsy Microsurgery 1994 15 11 768 772 7700137 10.1002/micr.1920151105

[7] Midha R Nerve transfers for severe brachial plexus injuries: a review Neurosurg Focus 2004 16 05 E5 10.3171/foc.2004.16.5.6 15174825

[8] Bhandari P S Sadhotra L P Bhargava P Singh M Mukherjee M K Bhatoe H S Dorsal approach in spinal accessory to suprascapular nerve transfer in brachial plexus injuries: technique details Indian J Neurotrauma 2010 7 01 71 74

[9] Segal D Cornwall R Little K J Outcomes of spinal accessory-to-suprascapular nerve transfers for brachial plexus birth injury J Hand Surg Am 2019 44 07 578 587 30898464 10.1016/j.jhsa.2019.02.004

[10] Cools A M Declercq G A Cambier D C Mahieu N N Witvrouw E E Trapezius activity and intramuscular balance during isokinetic exercise in overhead athletes with impingement symptoms Scand J Med Sci Sports 2007 17 01 25 33 16774650 10.1111/j.1600-0838.2006.00570.x

[11] Grossman J A Ruchelsman D E Schwarzkopf R Iatrogenic spinal accessory nerve injury in children J Pediatr Surg 2008 43 09 1732 1735 18779017 10.1016/j.jpedsurg.2008.04.029

[12] Lim J Y Lee J S Mun B M Kim T H A comparison of trapezius muscle activities of different shoulder abduction angles and rotation conditions during prone horizontal abduction J Phys Ther Sci 2015 27 01 97 100 25642047 10.1589/jpts.27.97 PMC4305608

[13] Chan G Y Nonato L G Chu A Raghavan P Aluru V Silva C T Motion Browser: visualizing and understanding complex upper limb movement under obstetrical brachial plexus injuries IEEE Trans Vis Comput Graph 2019 26 01 981 990 10.1109/TVCG.2019.2934280 31449022

[14] Bertelli J A Ghizoni M F Transfer of the accessory nerve to the suprascapular nerve in brachial plexus reconstruction J Hand Surg Am 2007 32 07 989 998 17826551 10.1016/j.jhsa.2007.05.016

[15] Emamhadi M Alijani B Andalib S Long-term clinical outcomes of spinal accessory nerve transfer to the suprascapular nerve in patients with brachial plexus palsy Acta Neurochir (Wien) 2016 158 09 1801 1806 27383201 10.1007/s00701-016-2886-1

[16] Garg R Merrell G A Hillstrom H J Wolfe S W Comparison of nerve transfers and nerve grafting for traumatic upper plexus palsy: a systematic review and analysis J Bone Joint Surg Am 2011 93 09 819 829 21543672 10.2106/JBJS.I.01602

[17] Tung T H Mackinnon S E Nerve transfers: indications, techniques, and outcomes J Hand Surg Am 2010 35 02 332 341 20141906 10.1016/j.jhsa.2009.12.002

[18] Bahm J Upper limb multifactorial movement analysis in brachial plexus birth injury J Brachial Plex Peripher Nerve Inj 2016 11 01 e1 e9 28077954 10.1055/s-0036-1579762 PMC5152696

[19] Heuberer P Kranzl A Laky B Anderl W Wurnig C Electromyographic analysis: shoulder muscle activity revisited Arch Orthop Trauma Surg 2015 135 04 549 563 25720847 10.1007/s00402-015-2180-3

[20] Raghavan P Santello M Gordon A M Krakauer J W Compensatory motor control after stroke: an alternative joint strategy for object-dependent shaping of hand posture J Neurophysiol 2010 103 06 3034 3043 20457866 10.1152/jn.00936.2009 PMC2888236

[21] Sheffler L C Lattanza L Sison-Williamson M James M A Biceps brachii long head overactivity associated with elbow flexion contracture in brachial plexus birth palsy J Bone Joint Surg Am 2012 94 04 289 297 22336968 10.2106/JBJS.J.01348 PMC3273876

[22] Tse R Marcus J R Curtis C G Dupuis A Clarke H M Suprascapular nerve reconstruction in obstetrical brachial plexus palsy: spinal accessory nerve transfer versus C5 root grafting Plast Reconstr Surg 2011 127 06 2391 2396 21617471 10.1097/PRS.0b013e3182131c7c

[23] Abdouni Y A Checoli G F Salles H C da Costa A C Chakkour I Fucs P MMB Assessment of the results of accessory to suprascapular nerve transfer Acta Ortop Bras 2018 26 05 332 334 30464716 10.1590/1413-785220182605193532 PMC6220659

[24] Ruchelsman D E Ramos L E Alfonso I Price A E Grossman A Grossman J A Outcome following spinal accessory to suprascapular (spinoscapular) nerve transfer in infants with brachial plexus birth injuries Hand (N Y) 2010 5 02 190 194 19882190 10.1007/s11552-009-9236-1 PMC2880671

